# Inhibition of autophagy enhances the selective anti-cancer activity of tigecycline to overcome drug resistance in the treatment of chronic myeloid leukemia

**DOI:** 10.1186/s13046-017-0512-6

**Published:** 2017-03-10

**Authors:** Ziyuan Lu, Na Xu, Bolin He, Chengyun Pan, Yangqing Lan, Hongsheng Zhou, Xiaoli Liu

**Affiliations:** Department of Hematology, Nanfang Hospital, Southern Medical University, 1838 Guangzhou Da Dao North, Guangzhou, 510515 China

**Keywords:** Tigecycline, Autophagy, Energy metabolism, Chronic myeloid leukemia, Drug resistance, Mitochondrial biogenesis

## Abstract

**Background:**

Drug resistance and disease progression are still the major obstacles in the treatment of chronic myeloid leukemia (CML). Increasing researches have demonstrated that autophagy becomes activated when cancer cells are subjected to chemotherapy, which is involved in the development of drug resistance. Therefore, combining chemotherapy with inhibition of autophagy serves as a new strategy in cancer treatment. Tigecycline is an antibiotic that has received attention as an anti-cancer agent due to its inhibitory effect on mitochondrial translation. However, whether combination of tigecycline with inhibition of autophagy could overcome drug resistance in CML remains unclear.

**Methods:**

We analyzed the biological and metabolic effect of tigecycline on CML primary cells and cell lines to investigate whether tigecycline could regulate autophagy in CML cells and whether coupling autophagy inhibition with treatment using tigecycline could affect the viabilities of drug-sensitive and drug-resistant CML cells.

**Results:**

Tigecycline inhibited the viabilities of CML primary cells and cell lines, including those that were drug-resistant. This occurred via the inhibition of mitochondrial biogenesis and the perturbation of cell metabolism, which resulted in apoptosis. Moreover, tigecycline induced autophagy by downregulating the PI3K-AKT-mTOR pathway. Additionally, combining tigecycline use with autophagy inhibition further promoted the anti-leukemic activity of tigecycline. We also observed that the anti-leukemic effect of tigecycline is selective. This is because the drug targeted leukemic cells but not normal cells, which is because of the differences in the mitochondrial biogenesis and metabolic characterization between the two cell types.

**Conclusions:**

Combining tigecycline use with autophagy inhibition is a promising approach for overcoming drug resistance in CML treatment.

## Background

Chronic myeloid leukemia (CML) is a myeloproliferative neoplasm companied with the BCR-ABL fusion gene, which is encoded by the Philadelphia chromosome [1]. The BCR-ABL fusion protein plays a key role in CML leukemogenesis by activating its downstream signaling pathway of survival and proliferation [2, 3]. Imatinib is a targeted competitive inhibitor of BCR-ABL tyrosine kinase that has changed the clinical treatment and prognosis of CML. Other tyrosine kinase inhibitors (TKIs) such as dasatinib and nilotinib have a higher anti-leukemic activity and are associated with fewer side effects. However, acquired resistance to TKIs is one of the main obstacles to effective CML treatment and is involved in gene amplification of ABL tyrosine kinase point mutations [4]. Patients with these ABL tyrosine kinase point mutations generally have a poor prognosis and a higher mortality rate.

The survival, proliferation and drug resistance property of leukemic cells are mainly dependent on their crosstalk with the bone marrow microenvironment [5]. In addition, hypoxia and nutrient deficiency are characteristics of the microenvironments of cancers [6]. Under such hostile surroundings, leukemic cells adapt dynamically by adjusting their metabolic phenotype. In order to maintain the energy balance between ATP production and the need for excessive proliferation and survival, cancer cells develop a novel metabolic phenotype that is more dependent on glycolysis than on mitochondrial oxidative phosphorylation (OXPHOS). This phenomenon is known as Warburg effect [7]. In contrast, some researches have recently demonstrated that mitochondrial respiration still plays a vital role in the metabolic properties of cancer cells by reinforcing mitochondrial activity [8, 9]. In tumorigenesis, mitochondria are not only biosynthetic organelles involved in bioenergetic reactions, but they are also survival signaling mediators for the cancer cells. Therefore, understanding and targeting the mechanisms underlying mitochondrial function would be critical exploitable points in the development of cancer therapies.

Autophagy can be induced when cancer cells subjected to hypoxia, poor nutritional conditions and chemotherapy to allow cancer cells adapt to the developmental microenvironment [10, 11]. Inhibition of tyrosine kinase by TKIs not only results in cell death but also causes the induction of autophagy in CML cells. Importantly, inhibition of autophagy by pharmacological inhibitors (chloroquine and bafilomycin A) or knockdown of the autophagy genes ATG5 and ATG7 in CML cells could potentiate TKIs-induced cell apoptosis in CML cells [12]. These researches demonstrated that inhibition of autophagy could improve the treatment efficiency of TKIs and combining of autophagy inhibition with other anti-cancer drug may be more efficient treatment to CML cells. Currently, it has been shown that the antibiotic tigecycline has an interesting “side effect” of inhibiting mitochondrial translation in cancer cells, which results in the killing of cancer cells, as well as cancer stem cells [13]. The observations challenged the traditional concept that cancer treating like an infectious disease with less toxicity. Based on this premise, we hypothesized that tigecycline could be used as an anticancer drug, as it induces significant apoptosis of CML cells companied with autophagy. Moreover, inhibition of autophagy could enhance the anti-leukemic effect of tigecycline and reverse drug resistance to TKI therapy which mainly attributable to ABL tyrosine kinase point mutations.

## Methods

### Cell lines and cultures

BCR-ABL positive KBM5 cells and K562 cells were used as imatinib-sensitive cell lines. On the other hand, KBM5 cells with T315I mutation (KBM5-STI cells), which were provided by Prof. Michael Andreeff (Section of Molecular Hematology and Therapy, Department of Leukemia, The University of Texas M.D. Anderson Cancer Center, Houston, TX, USA), were used as an imatinib-resistant cell line. Thirteen primary CML cells were obtained from eight newly diagnosed patients (cases at chronic phase without any mutations) and five refractory patients (2 cases with T315I mutation, 3 cases without any mutations) after obtaining written informed consent. Eight healthy donors were selected by matching their ages and sex to those of the CML patients. The study was approved by the Institutional Review Board of Nanfang Hospital (Guangzhou, China).

### Cell viability assay

Cell viability assay was performed using Cell Counting Kit-8 (CCK8; CK40; Dojingdo, Kumamoto, Japan). Cells were seeded in 96-well plates and then exposed to tigecycline (Hisun Pharmaceutical Company, Fuyang, China) and/or chloroquine (CQ) (14774; Cell Signaling Technology, Danvers, MA) at the indicated concentrations for 48 h. The cells were incubated with CCK-8 solution for 2 h, after which absorbance was measured at 450 nm using a SpectraMax Plus microplate reader (Molecular Devices, Sunnyvale, CA, USA).

### Flow cytometry analysis

After stimulation with tigecycline and/or CQ, the cells were subjected to flow cytometry experiments. An annexin V-FITC and PI apoptosis detection kit (AD10, Dojingdo) was used for apoptosis assay according to the manufacturer’s instructions. A mitochondrial membrane potential assay kit (12664, Cell Signaling Technology, Danvers, MA, USA) was used to detect mitochondrial membrane potential in the CML cells using the fluorescent dye JC-1.When JC-1 permeates into intact mitochondria, it aggregates to orange-red fluorescence. However, JC-1 infiltrates into impaired mitochondria, the dye changed into green fluorescence. The relative ratio of the green/red fluorescence is therefore used as an indicator of mitochondrial membrane potential. The generation of reaction oxygen species (ROS) was monitored after the cells were stained with a fluorometric dye using a Fluorometric Intracellular ROS Kit (MAK142; Sigma-Aldrich, St. Louis, MO, USA) according to the manufacturer’s instructions. Furthermore, mitochondrial mass was measured using Mito Green Probe (KGMP0072, Keygen Biotech, Nanjing, China) after incubating the cells at 37 °C for 30 min. All the assays mentioned above were digitized using a FACSCalibur flow cytometer (Becton-Dickinson, Franklin Lakes, NJ, USA).

### Measurement of oxygen consumption rate (OCR) and extracellular acidification rate (ECAR)

OCR and ECAR were measured using Seahorse XF24 Flux Analyzer (Seahorse Bioscience, Shanghai, China). Briefly, the cells were plated on XF24 cell culture plates coated with Cell-Tak solution (1 mg/mL; MAP-O4012; ACRO Biosystems, Newark, DE, USA). K562, KBM5, and KBM5-STI cells were seeded at 250,000 cells/well, 300,000 cells/well, and 300,000 cells/well, respectively, whereas primary CML cells were plated at 300,000 cells/well. The cells were seeded in each well in 100 μL of XF Assay Medium (102353–100, Seahorse Bioscience) supplemented with 1 mM pyruvate, 2 mM glutamine, and 10 M glucose for OCR analysis, or 1 mM glutamine for ECAR measurement. Cell Mito Stress Test Kit (103015–100, Seahorse Bioscience) and Glycolysis Stress Test Kit (103020–100, Seahorse Bioscience), which contained the relevant compounds for each assay, were used to measure OCR and ECAR, respectively. Oligomycin, carbonyl cyanide 4-(trifluoromethoxy)phenylhydrazone (FCCP), and a mixture of rotenone and antimycin A were used for the OCR analysis, whereas glucose, oligomycin, and 2-deoxyglucose (2-DG) were used for the ECAR analysis. The compounds were serially injected into the cells to measure mitochondrial respiration and glycolysis function, respectively.

### Western blotting

Western blotting was performed as before [8]. Briefly, proteins were separated by sodium dodecyl sulfate polyacrylamide gel electrophoresis (SDS-PAGE), transferred to nitrocellulose membranes, and sequentially incubated overnight with primary antibodies at 4 °C. After incubation with secondary antibodies, signals were visualized using a chemiluminescent detection reagent (WBKLS0500; Millipore, Billerica, MA, USA). Before the analysis of cytochrome c, the cytosolic and mitochondrial sub-cellular fractions needed were separated using a mitochondria isolation kit (C3601; Beyotime Biotechnology, Shanghai, China). The western blotting analysis was conducted using the following primary antibodies: anti-cytochrome c (11940), anti-cleaved-caspase-9 (32539), anti-cleaved-caspase-3 (13847), anti-LC3B (51520), and anti-p62 (91526), which were obtained from Abcam (Cambridge, UK); and p-AKT (Ser473, 4060), p-AKT (Thr308, 13038), AKT (9272), p-mTOR (Ser2448, 5536), p-P70S6K (Thr389, 9234), p-4E-BP1 (Thr37/46, 2855), and anti-ATG5 (12994), which were obtained from Cell Signaling Technology; and anti-Cox-1(19998), anti-Cox-2(19999) and anti-Cox-4 (69359), which were obtained from Santa Cruz Biotechnology (California, USA).

### Fluorescence and confocal microscopy

After treatment with tigecycline, CQ, or 3-methyladenine (3-MA) (9281, Sigma-Aldrich), the CML cells were prepared for the autophagy detection experiment. An autophagy detection kit (139484, Abcam) was optimized and used for the detection of autophagy in the CML cells by fluorescent scanning confocal microscopy (Olympus FV10i; Olympus, Tokyo, Japan).

### Transmission electron microscopy

The cells were stained with uranyl acetate and lead citrate on a microtome (Leica Ultracut; Leica, Wetzlar, Germany) and examined with a transmission electron microscope (Hitachi H-7650; Hitachi, Tokyo, Japan) at an accelerating voltage of 60 KV. Micrographs were taken at × 7,000 or × 20,000 magnification.

### Quantitative PCR (QPCR)

Mitochondrial DNAs (mtDNAs) were extracted from the fresh bone marrow mononuclear cells obtained from the CML patients and healthy donors and stored at −20 °C until use. Relative mtDNA copy number and the mRNA levels of Cox-1, Cox-2 and Cox-4 were determined by qPCR method. The primer sequences used were as follows: forward primer (Cox-1-F) 5’-CTATACCTATTATTCGGCGCATGA-3’and reverse primer (Cox-1-R) 5’- CAGCTCGGCTCGAATAAGGA-3’ for Cox-1; forward primer (Cox-2-F) 5’- CTGAACCTACGAGTACACCG-3’ and reverse primer (Cox-2-R) 5’- TTAATTCTAGGACGATGGGC-3’ for Cox-2; forward primer (Cox-4-F) 5’- GCCATGTTCTTCATCGGTTTC-3’ and reverse primer (Cox-4-R) 5’- GGCCGTACACATAGTGCTTCTG-3’ for Cox-4; forward primer (18 s-F) 5’- AGGAATTGACGGAAGGGCAC-3’ and reverse primer (18 s-R) 5’- GGACATCTAAGGGCATCACA-3’for 18S; forward primer (ND1-F) 5’-CCC TAA AAC CCG CCA CAT CT-3’ and reverse primer (ND1-R) 5’-GAG CGA TGG TGA GAG CTA AGG T-3’ for ND1; and forward primer (human globulin (HGB)-F) 5’-GTG CAC CTG ACT CCT GAG GAG A-3’ and reverse primer (HGB-R) 5’-CCT TGA TAC CAA CCT GCC CAG-3’ for HGB. QPCR reactions were performed on an ABI Prism 7900 sequence detection system (Applied Biosystems, Foster City, CA). The relative abundance of a transcript was represented by the threshold cycle of amplification (CT). The comparative CT method was calculated per the manufacturer's instructions. The expression level of Cox-1 relative to the baseline level was calculated as 2^–ΔC^_T_^(Cox-1)^, where ∆CT is (average Cox-1 C_T_ – average 18 s C_T_) and is CT (average C_T_ -treated sample – average C_T_ -untreated sample. The calculation methods of expression levels of Cox-2, Cox-4 and ND1 are the same as that of Cox-1. For the calculation method of expression level of ND1, the reference one is HGB.

### Small interfering RNA transfection

Cells were transfected with siRNA (ATG5-F, 5’ CAACTTGTTTCACGCTATATCAGG; ATG5-R, 5’ CACTTTGTCAGTTACCAACGTCA) designed by Guangzhou Land Bio Co. Ltd. (Guangzhou, China) using Lipofectamine® 2000 Transfection Reagent (11668; Invitrogen, Carlsbad, CA, USA) according the manufacturer’s instructions.

### Statistical analysis

GraphPad Prism 5 (GraphPad Software Inc., San Diego, CA, USA) was used to perform statistical analysis. The results have been expressed as mean ± standard deviations. Test of comparison between two groups were done using Student’s *t*-test (two tailed) and comparisons between multiple groups were done by one-way ANOVA. *P* values < 0.05 were considered statistically significant.

## Results

### Tigecycline reduced the viabilities of the primary CML cells and cell lines

**Fig. 1:**
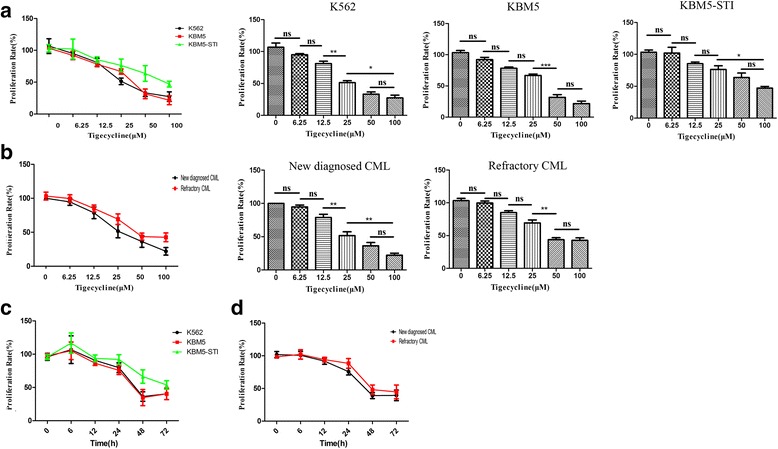
Tigecycline inhibits the proliferation of CML cells in dose- and time-dependent manners. (**a**, **c**) Viabilities of CML cell lines (K562, KBM5, and KBM5-STI) after treatment with different concentrations of tigecycline treatment in different time points. (**b**, **d**) Proliferations of primary CML cells obtained from newly diagnosed CML patients and refractory CML patients after treatment with different concentrations of tigecycline in different time points. Error Bars: SD of 3 independent experiments;* *P* < 0.05, **P < 0.01, ***P < 0.001

Initially, we determined whether tigecycline could inhibit the viability of CML cells. We chose K562 and KBM5 cell lines as imatinib-sensitive phenotypes, while KBM5 cells with T315I mutations (KBM5-STI cells) were the imatinib-resistant genotype. The cells were similarly treated with increasing concentrations of tigecycline (6.25–100 μM) for 48 h. The half maximal inhibitory concentration (IC50) of tigecycline ranged from 51.40 to 86.07 μM against the three leukemia cell lines (Fig. 1a). Therefore, in order to standardize the experimental conditions, we used tigecycline at a concentration of 50 μM in subsequent experiments. It was noted that the inhibitory action of tigecycline was dose- and time-dependent and occurred irrespective of the cytogenetic mutation status of the cells (Fig. 1a, c). Moreover, the inhibitory effects of tigecycline were equally observed in primary CML cells obtained from the different patients (Fig. 1b, d).

### Tigecycline inhibited mitochondrial biogenesis in the CML cells

**Fig. 2:**
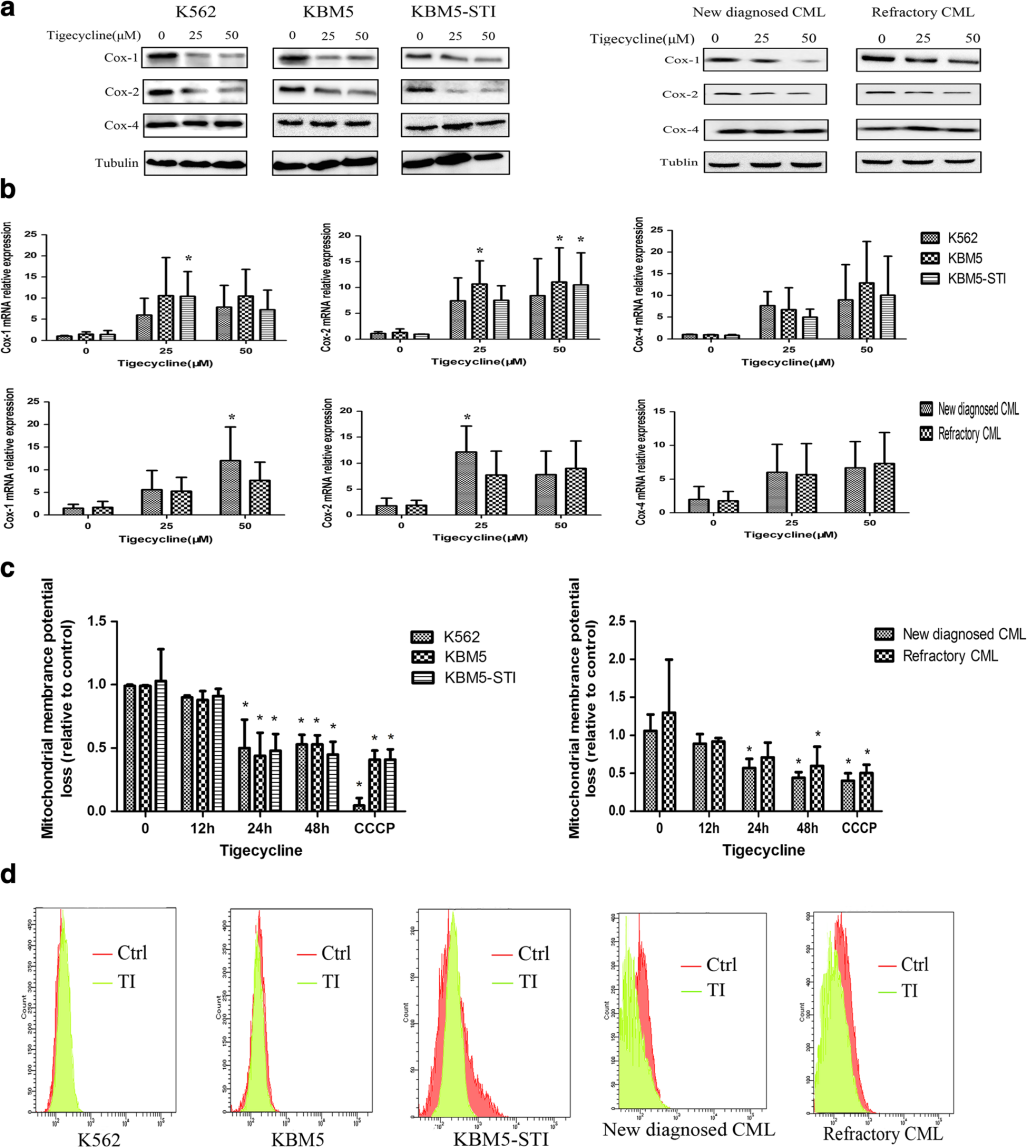
Tigecycline suppresses mitochondrial biogenesis in CML cell lines and primary cells. (**a**) Effects of increasing concentrations of tigecycline on the protein levels of cytochrome c oxidase (Cox)-1, Cox-2, and Cox-4 in CML cell lines and primary cells. Tubulin was used as the reference protein in the western blotting. All the cells were cultured with tigecycline for 48 h before the experiments were conducted. (**b**) The relative mRNA levels of Cox-1, Cox-2, and Cox-4 in CML cells after treatment with tigecycline. (**c**) Evaluation of the mitochondrial membrane potential of tigecycline-treated CML cells using JC-1 staining and flow cytometry. Carbonyl cyanide 3-chlorophenylhydrazone (CCCP) was used as the positive control. (**d**) Reactive oxygen species (ROS) levels in the CML cells were measured by flow cytometry. Ctrl, control; TI, tigecycline-treated cells. *P < 0.05

Molecular disruption of mitochondrial biogenesis or OXPHOS could be the target of tigecycline [13]. To understand the mechanism underlying the anti-leukemic effect of tigecycline, mitochondrial function experiments were performed. In the first set of experiments, we measured the levels of cytochrome c oxidase-1, 2, and 4 (Cox-1, 2, and 4) by western blotting and quantitative polymerase chain reaction (qPCR) after tigecycline treatment. Mitochondria have an independent genome encoding system that is responsible for two rRNAs, 22 t-RNAs, and 13 of the 90 proteins in the mitochondrial respiratory chain [14]. Cox-1 and Cox-2 are the representative mitochondrial encode proteins, while Cox-4 is encoded by a nuclear genome [15]. After tigecycline stimulation, our data showed that Cox-1 and Cox-2 protein levels significantly decreased as compared to that of Cox-4 (Fig. 2a). However, reductions in Cox-1 and Cox-2 protein levels did not result in reductions in their respective mRNA levels in the same cells (Fig. 2b). In addition, these changes were observed in the primary CML samples (Fig. 2a, b). This suggests that the anti-leukemic activity of tigecycline is implicated in the inhibition of mitochondrial protein translation.

Many important proteins in the mitochondrial respiratory chain are encoded by the mitochondrial genome. Mitochondrial membrane potential, which is the strong electrochemical proton gradient across the inner membrane, is generated by the mitochondrial respiratory chain. Here, a sensitive cationic and lipophilic JC-10 fluorescent probe was used to monitor mitochondrial membrane potential changes in the cells based on the presence of the JC-10 dye. In the flow cytometry analysis, the mitochondrial membrane potentials of the cell lines and primary CML cells began to decrease at different degree after 24 h of stimulation with tigecycline as the effect of the mitochondrial membrane potential disruptor carbonyl cyanide 3-chlorophenylhydrazone (CCCP) (Fig. 2c). These data indicate that mitochondrial membrane depolarization precedes loss of viability in leukemic cells exposed to tigecycline.

Mitochondria are the factory of energy supply where ATP is synthesized. It has equally important roles such as reactive oxygen species (ROS) production. To investigate the involvement of oxidative stress in tigecycline-induced mitochondrial impairment, intracellular ROS levels were measured. Surprisingly, tigecycline did not cause an increase in ROS levels in any of the cell lines and patient samples during the treatment period (Fig. 2d). We postulated that tigecycline produced inhibitory effects possibly through a novel mechanism that is distinct from the inhibition of mitochondrial function.

### Tigecycline suppressed mitochondrial stress and glycolysis levels in the CML cells

**Fig. 3:**
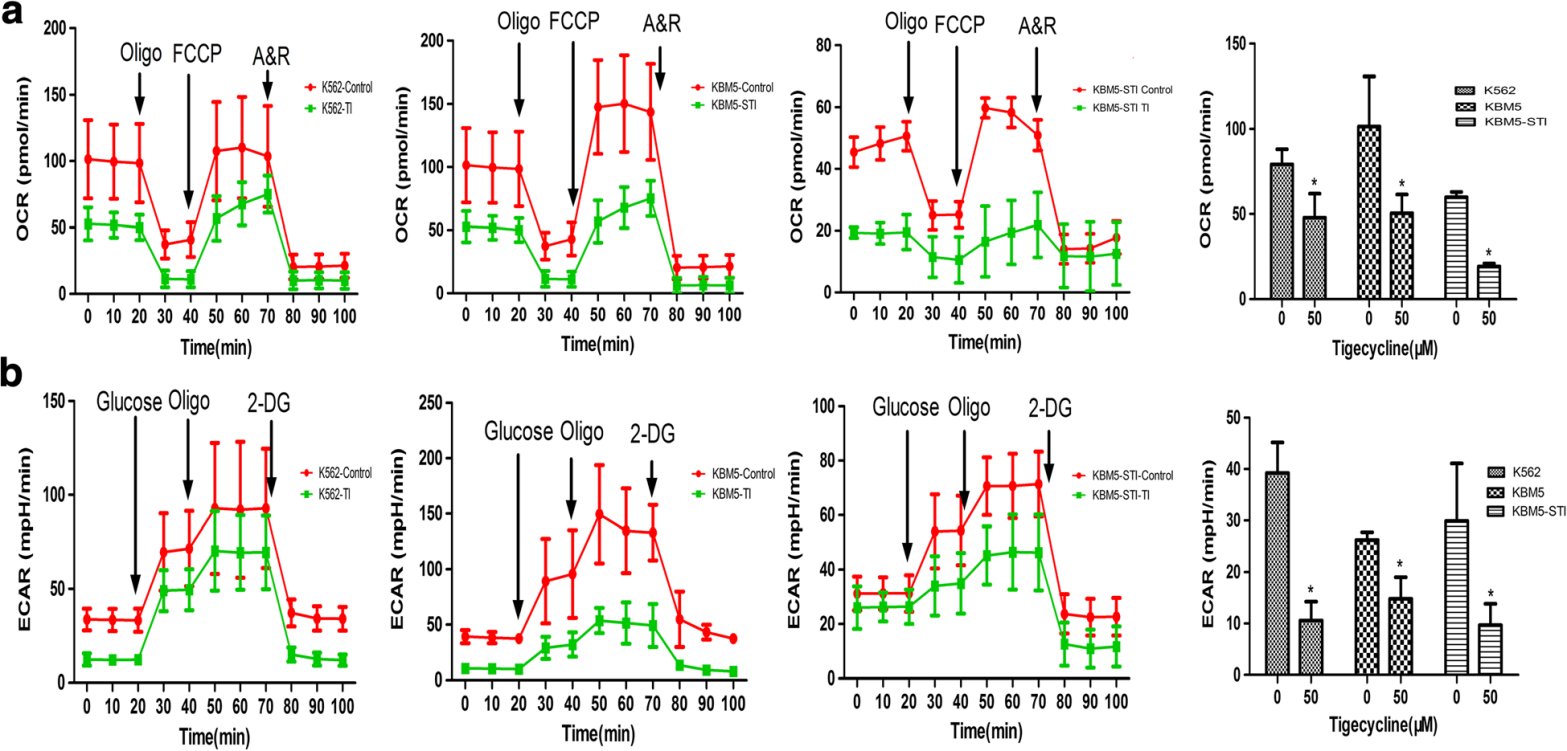
Tigecycline inhibits energy metabolism in CML cells. (**a**) Mitochondrial respiration of the CML cells was measured before and after treatment with tigecycline, and is represented by the oxygen consumption rate (OCR) curve and values. Oligo, oligomycin; A&R, antimycin A and rotenone. (**b**) Effect of tigecycline on glycolysis in the CML cells. Glycolysis capacities of the CML cells are shown by the extracellular acidification rate (ECAR) curves and values. Oligo, oligomycin. *P < 0.05

Damaged mitochondria generate excessive amounts of harmful substances, which lead to disturbance of mitochondrial bioenergy homeostasis and eventually programmed cell death [16]. Therefore, we monitored the levels of two main cellular ATP generation mechanisms, mitochondrial respiration and glycolysis, in the context of tigecycline strong stimulation. In the real-time assay, we measured the levels of mitochondrial stress and glycolysis and reported them as oxygen consumption rate (OCR) and extracellular acidification rate (ECAR), respectively. As illustrated in Fig. 3a, there was a significant decrease in baseline OCR in the tigecycline-treated cells. In the presence of tigecycline, the ATP synthase inhibitor oligomycin decreased OCR markedly. However, after stimulation with carbonyl cyanide-4-(trifluoromethoxy) phenylhydrazone (FCCP), the OCR of the tigecycline-treated cells did not increase to the peak value like that of the control cells did. Moreover, the tigecycline-treated cells were sensitive to the inhibitory effect of antimucin A/rotenone (A/R) in the OCR assay. In addition, the OCR values of the tigecycline-treated cell lines were significantly lower than those of the control cells were. Furthermore, we observed that the glycolysis level in the control cells, as measured in the ECAR assay, increased due to the serial addition of glucose and oligomycin to the culture medium. However, this increase in glycolysis was prevented by 2-deoxy-D-glucose (2-DG), which is a glycolysis inhibitor. We however noted that the abovementioned changes were unremarkable in the presence of tigecycline. In addition, ECAR decreased significantly in the tigecycline-treated cells (Fig. 3b). These data demonstrate that tigecycline has strong inhibitory effects on mitochondrial respiration and glycolysis capacity, which are processes in mitochondrial bioenergy homeostasis.

### Tigecycline caused apoptosis of the CML cells by activating the cytochrome c/caspase-9/caspase-3 pathway

**Fig. 4:**
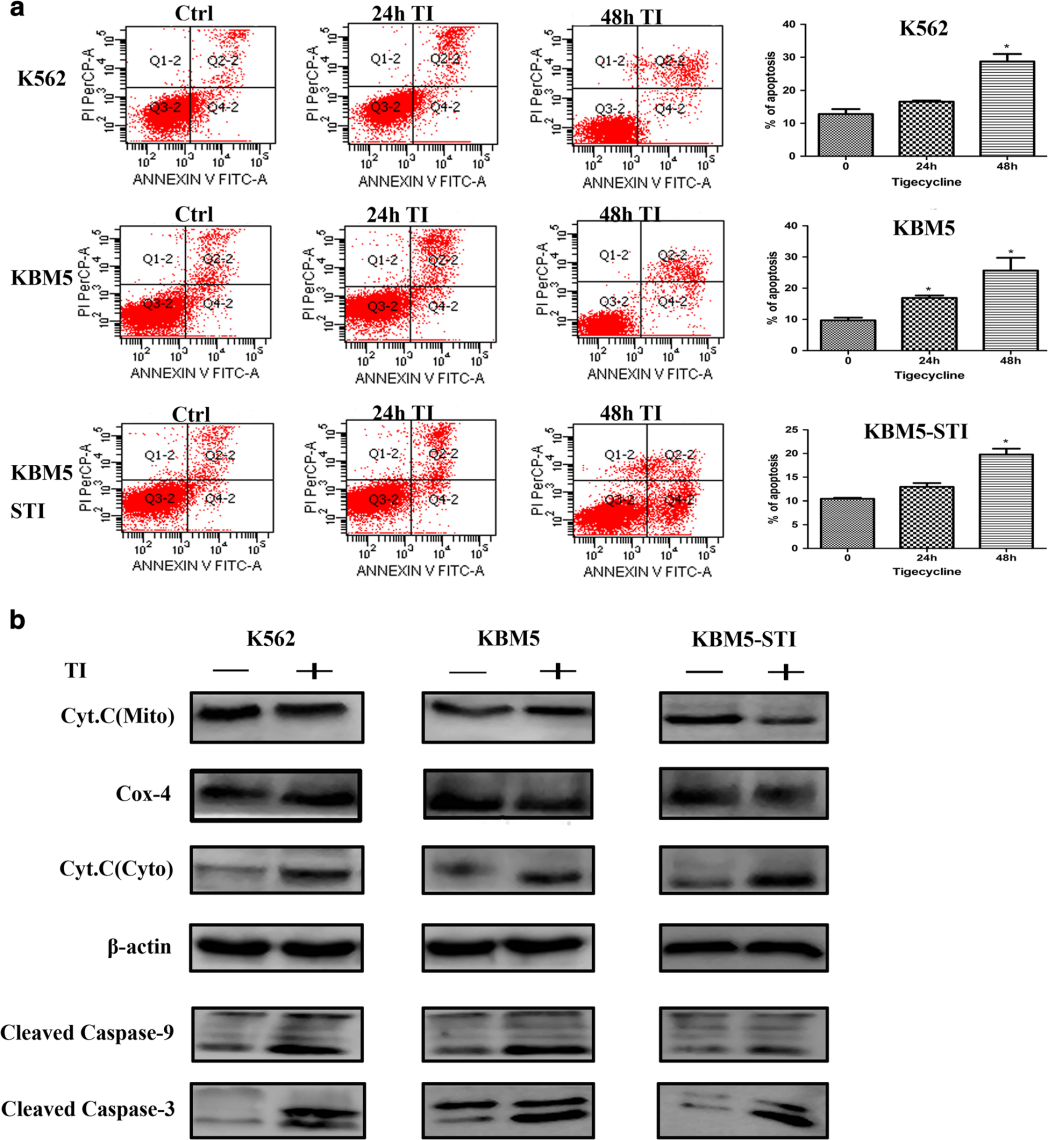
Tigecycline causes apoptosis of CML cells by activating the cytochrome c/caspase-9/caspase-3 signaling pathway. (**a**) Apoptosis assay of the CML cells in response to stimulation with tigecycline. Left panel: a representative flow cytometry plots for CML cells stained with annexin V-FITC/PI-stained. Right panel: percentage apoptosis of the CML cells. Apoptosis was defined as the percentage of annexin V-positive cells. (**b**) Western blot analyses of cytochrome c, cleaved caspase-9, and caspase-3 protein levels. Cyto.C (Mito), cytochrome c protein in the mitochondria; Cyto.C (Cyto), cytochrome c protein in the cytoplasm. Cytochrome c oxidase-4 and β-actin were used as the reference proteins for the analyses of mitochondrial and cytoplasmic proteins, respectively. *P < 0.05

Mitochondrial membrane depolarization and accumulation of ROS are features of apoptosis. Flow cytometry using Annexin V combined with PI was used to assay the apoptotic effect of tigecycline on the CML cell lines. Apoptosis was only slightly observed in the KBM5 cells after 24 h of treatment with tigecycline; however, no apoptosis was observed in the K562 and KBM5-STI cells, and the effects enlarged significantly after 48 h of exposure to the drug in all the cell lines (Fig. 4a). When apoptosis is initiated, cytochrome c, which is a pro-apoptotic protein and part of the respiratory chain, is released from the mitochondrial inner membrane into the cytoplasm to trigger apoptosome formation. This results in the activations of caspase-9 and the downstream effector caspase-3, which ultimately lead to apoptosis [17, 18]. Therefore, we used western blotting to detect cytochrome c/caspase-9/caspase-3 protein levels pre- and post-treatment. There were no obvious changes in cytochrome c, caspase-9, and caspase-3 levels in the untreated cells. However, exposure to tigecycline resulted in increased cytochrome c levels in the cytoplasm of the treated cells. Furthermore, in all the cell lines, we observed that the levels of downstream sensors of cytochrome c, cleaved caspase-9, and caspase-3 in the tigecycline-treated cells were greater than the levels of respective proteins in the control cells (Fig. 4b). Collectively, our data indicate that there was a redistribution of cytochrome c and activations of caspase-9 and caspase-3 in response to the pro-apoptotic effect of tigecycline in the CML cell lines.

### Tigecycline induced autophagy in the CML cells by downregulating the AKT-mTOR signaling pathway

**Fig. 5:**
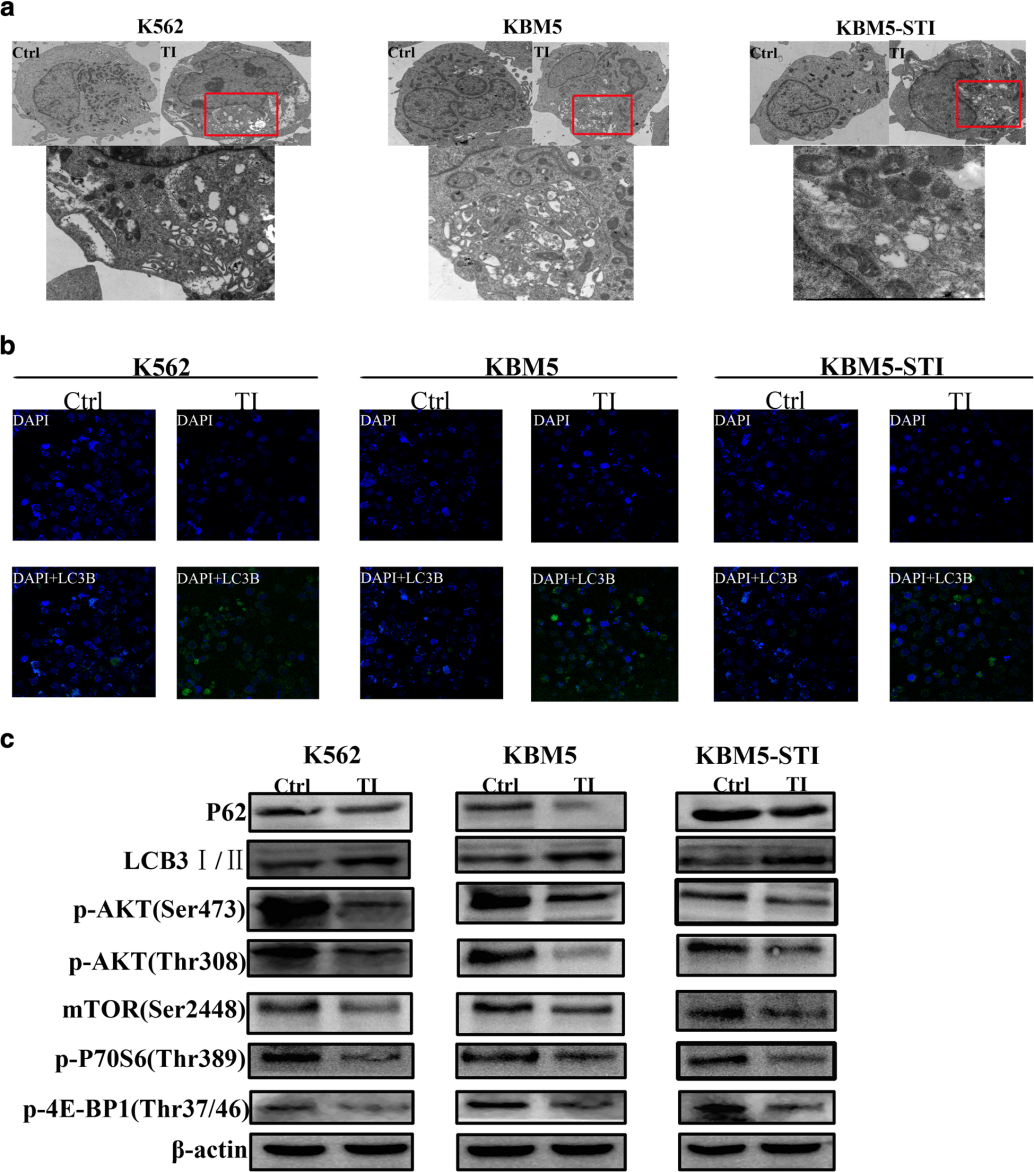
Tigecycline induces autophagy of CML cells by downregulating the PI3K-ATK-mTOR signaling pathway. (**a**) Autophagic vacuoles were measured by transmission electron microscopy. Upper panel: autophagic vacuoles in CML cells with and without tigecycline treatment. Lower panel: amplified image of autophagic vacuoles in tigecycline-treated CML cells. (**b**) Confocal microscopy analysis of autophagy. Blue spots indicate nuclei stained with 4',6-diamidino-2-phenylindole (DAPI). Green spots indicate autophagic vacuoles stained with LC3B dye. (**c**) Western blot analysis to evaluate the levels of autophagy related protein P62 and LC3B, and mTOR and its upstream regulator AKT, and downstream sensors P70S6 and 4E-BP1

Recently, intense attention has been paid to the roles of autophagy in cancer development and chemotherapy resistance [19–21]. Many common anticancer drugs induce not only apoptosis but also autophagy in cancer cells. Therefore, we investigated whether tigecycline could induce autophagy in the CML cells. Using transmission electron microscopy, a standard autophagy detection method, we found more typical double-membrane autophagosomes in the tigecycline-treated CML cells than in the control cells (Fig. 5a).

To confirm tigecycline-induced autophagy, we chose a novel permeable and specific fluorescent probe for monitoring autophagy in live cells by confocal microscopy. When autophagy occurs in cells, the green fluorescent detection reagent is incorporated into the cells. Next, green spots which represent the autophagosome marker LC3 distribute throughout the cytoplasm or accumulate in the perinuclear region of the cells. The confocal microscopy showed an appreciable increase in green fluorescent spots either in the cytoplasm or surrounding blue fluorescent nuclei in the K562, KBM5, and KBM5-STI cells after treatment with tigecycline. However, the untreated cells did not show an obvious green fluorescence (Fig. 5b).

Furthermore, we investigated if there was autophagic flux in the tigecycline-treated cells. As the well-known and vital proteins in autophagic flux, SQSTM1 (p62) and LC3BI/II were selected as the representative proteins of autophagy in the western blotting assay. The assay showed a decrease in p62 protein levels and an increase in LC3BII protein levels in the tigecycline-treated CML cells (Fig. 5c). Taken together, our results indicate that tigecycline caused autophagy in the CML cell lines.

PI3K-AKT-mTOR signal pathway is activated by several stimuli involved in cell proliferation, survival, and metabolism, as well as autophagy. mTOR is a critical mediator in autophagy induction. When activated, mTOR (AKT and MAPK pathways) suppresses autophagy; however, its negative regulation (AMPK and p53 pathways) results in the promotion of autophagy [22]. Currently, the PI3K-AKT-mTOR pathway has been an attractive molecular target for novel anticancer therapies in many types of cancers. It was observed that tigecycline significantly inhibited this signaling pathway, as was evidenced by inhibition of the phosphorylations of AKT (Ser473 and Thr308) and mTOR (Ser2448), as well as their downstream protein P70S6 (Thr389) and 4E-BP1 (Thr37/46) (Fig. 5c). These results indicate that tigecycline can induce autophagy by inhibiting the PI3K-AKT-mTOR signaling pathway.

### Pharmacological inhibition or genetic ablation of autophagy enhanced tigecycline-induced death of CML cells

**Fig. 6:**
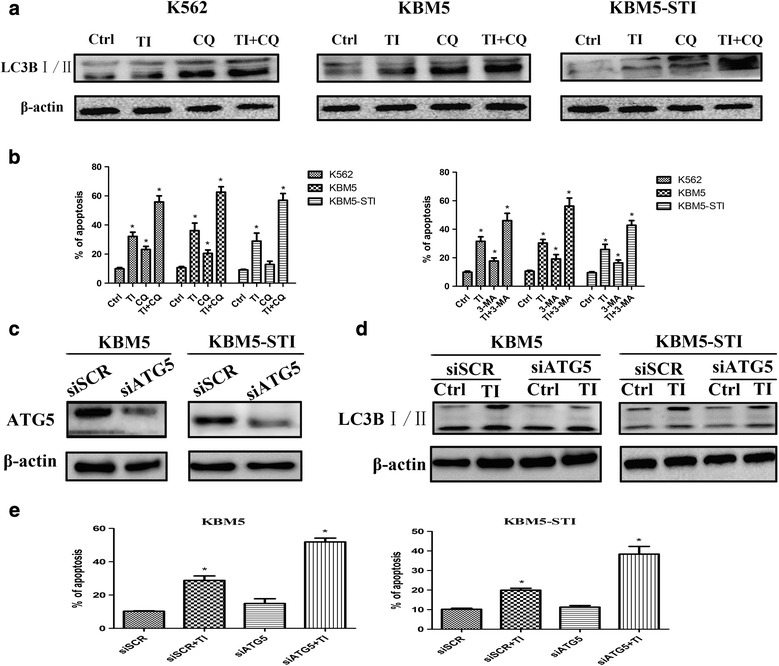
Combining tigecycline use with autophagy inhibition shows synergetic cytotoxicity against CML cells. (**a**) Protein level of LC3B in CML cells that were treated with tigecycline and/or chloroquine (CQ). (**b**) Assay of apoptosis of CMLcells exposed to tigecycline, CQ, 3-methyladenine(3-MA), or their combination treatment. (**c**) ATG5 protein levels in KBM5 and KBM5-STI cells that were transiently transfected with siATG5 and evaluated by western blotting. (**d**) LC3B protein levels in CML cells with and without ATG5 knockdown were measured after treatment with tigecycline. (**e**) Apoptotic assay of CML cells subjected to ATG5 silence after stimulation with tigecycline. *P < 0.05

We next sought to determine whether inhibition of autophagy would interfere with the inhibitory effect of tigecycline. Some studies have suggested that CQ suppresses autophagosomal fusion and degradation by inhibiting lysosomal hydrolases, which leads to apoptosis [23, 24]. Therefore, CQ can prevent the autophagy marker LC3BII from degrading and induce an increase in LC3BII protein levels. Due to its inhibitory effect on autophagy, CQ was selected as the autophagy blocker in this study. Our results showed that the protein level of LC3BII in the cells cultured with tigecycline and CQ was markedly higher than that in the cells treated with tigecycline or CQ alone (Fig. 6a). This suggested that CQ significantly inhibited tigecycline-induced autophagy. Notably, the flow cytometry analysis revealed that, the treatment with tigecycline combined with CQ resulted in a strong synergetic effect on apoptosis, which was higher than the effect of either agent alone (Fig. 6b). Furthermore, this synergistic effect was still observed when CQ was changed to 3-MA, which is also an autophagy inhibitor (Fig. 6b). In order to further investigate whether autophagy directly contributes to enhancing tigecycline-induced apoptosis, we used siRNA technology to target the key autophagy molecule ATG5 to ablate autophagy at the genetic level in the KBM5 and KBM5 ^T315I^ cell lines. As shown in Fig. 6c, the protein level of ATG5 in the ATG5 siRNA-transfected cells was significantly lower than that in the cells transfected with SCR siRNA. Moreover, tigecycline did not induce the conversion of LC3BI to LC3BIIin the siRNA-ATG5 cells (Fig. 6d). In the flow cytometry analysis, knockdown of ATG5 by siRNA markedly increased cell sensitivity to the inhibitory effect of tigecycline more than transfection with SCR siRNA did (Fig. 6e). Taken together, these results indicate that combining tigecycline use and autophagy inhibition could be an effective approach in CML treatment, especially since the synergistic effect of the two drugs was still observed irrespective of the ABL tyrosine kinase point mutation status of the cells.

### The selective anticancer effect of tigecycline is based on mitochondrial dependency and the metabolism type occurring in the tumor

**Fig. 7:**
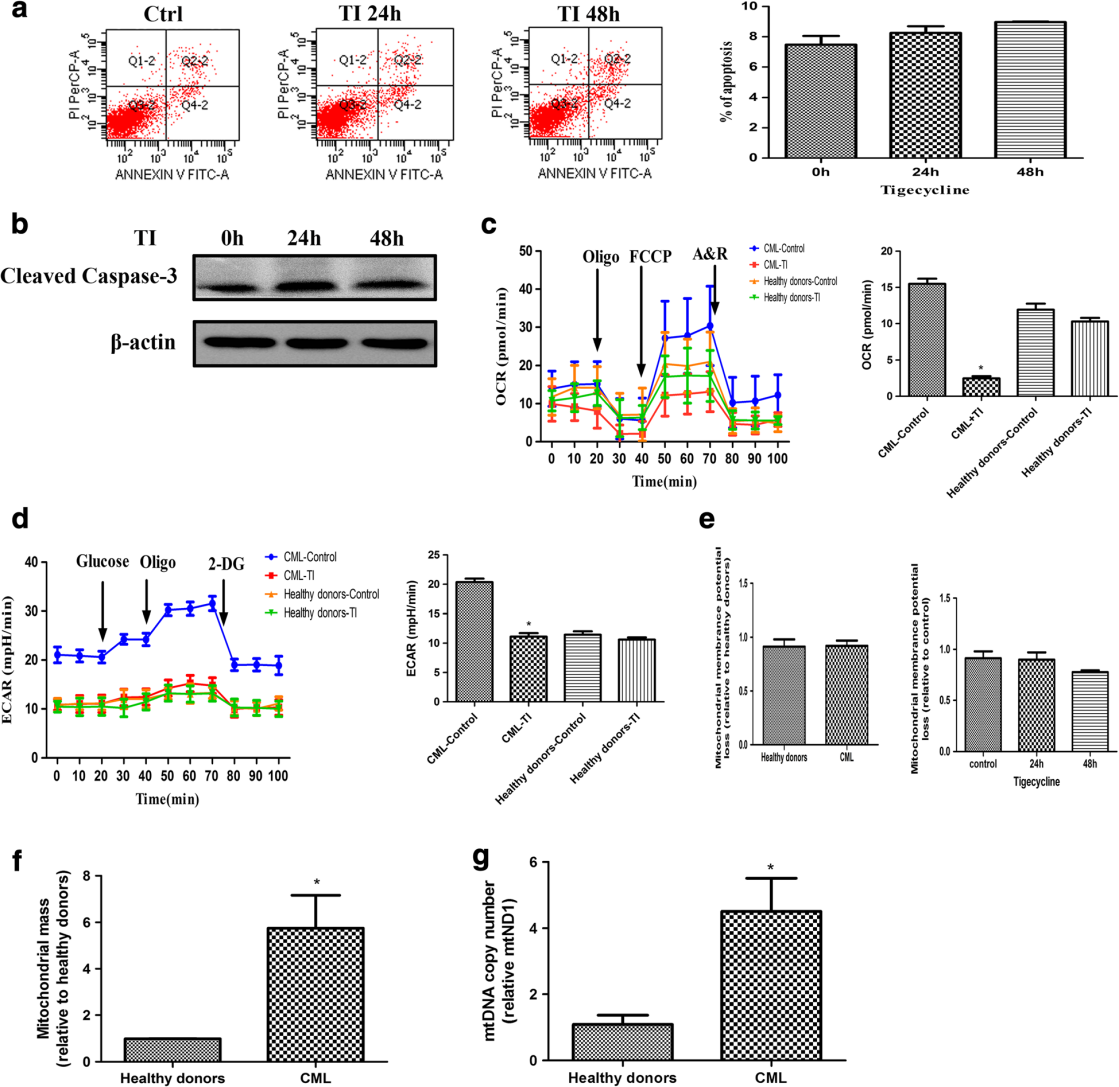
Tigecycline has no obvious inhibitory effect on normal cells. (**a**) Death of normal bone marrow mononuclear cells exposed to tigecycline in different time points were measured by flow cytometry. (**b**) Western blot analysis of cleaved caspase-3 protein level in normal cells. (**c**) Effect of tigecycline on the oxygen consumption rate (OCR) curves and values for CML cells and normal cells. (**d**) Analyses of the glycolysis capacity of leukemic and normal cells were performed before and after stimulation with tigecycline. (**e**) The mitochondrial membrane potentials of leukemic and normal cells before and after treatment with tigecycline. (**f**) Mitochondrial mass of normal cells and CML cells was measured by incubating the cells with MitoTracker® Green FM dye. (**g**) Mitochondrial DNA (mtDNA) copy number was analyzed by QPCR. *P < 0.05

As previously mentioned, we inferred that tigecycline may exert a powerful anticancer activity against CML cells, including those with poor prognosis markers. However, it is unknown whether tigecycline selectively targeted the CML cells. We used bone marrow mononuclear cells from healthy donors to study the effects of tigecycline on normal cells. Surprisingly, tigecycline did not trigger the death of the normal bone marrow mononuclear cells even after it was used at a relatively high concentration (Fig. 7a). In line with the results of the apoptosis study, tigecycline did not increase the protein level of cleaved caspase-3 in the normal cells (Fig. 7b). This suggests that tigecycline may be inactive against bone marrow mononuclear cells from healthy donors, which were representative of normal cells in the study.

We further studied the different effects of tigecycline on cancer cells and normal cells by measuring the metabolic characteristics of both cells using an extracellular flux analyzer to determine live cell OCR and ECAR. In the OCR curve, the bone marrow mononuclear cells from both CML patients and healthy donors showed similar levels of basal respiration; however, the maximal and minimal respiration levels were lower in the CML samples (Fig. 7c). On the other hand, in the glycolytic function assay, the ECAR level in the CML cells was significantly higher than that in the healthy cells (Fig. 7d). When the cells were treated with the same concentration of tigecycline, only the CML cells showed significant decreases in OCR and ECAR (Fig. 7c, d). It suggested that the CML cells were dependent on mitochondrial bioenergy functions, which may be a weakness of CML cells that tigecycline targeted.

Next, we assayed the mitochondrial characteristics of the mononuclear cells from the healthy donors and CML patients. Initially, there were no differences in the basal mitochondrial membrane potentials between the two groups of cells. In addition, there was no mitochondrial membrane depolarization in the normal cells even after 48 h of treatment with tigecycline (Fig. 7e). Furthermore, after using MitoTracker® Green FM dye to stain mitochondria irrespective of the mitochondrial membrane potential, we observed that mitochondrial mass was higher in the CML cells than in the normal cells (Fig. 7f). Finally, we evaluated the mtDNA copy number by qPCR using ND1 as the mtDNA gene and HGB as the nuclear gene [25]. The cells obtained from the CML patients had a significantly higher relative mtDNA content compared to those obtained from the normal subjects (Fig. 7g). Overall, these findings validate that leukemic cells display a greater mitochondrial dependence and a larger bioenergy reliance than normal cells do. In addition, the results explain why tigecycline shows a selective anticancer activity.

## Discussion

Although the major breakthough TKIs make, the emergence of drug resistance, which can occur in a mutant-dependent or mutant-independent manner, is still is a challenge in their use. The potential therapeutic targets in cases of drug resistance, which are based on differences between cancer cells and normal cells, remain to be explored. In tumorigenesis, glycolysis is not only an energy-production means but also a biosynthetic tool that supports cell survival and proliferation. Mounting evidence shows that abnormal dependence on glycolysis during energy production in cancer cells is well correlated to therapeutic resistance in cancer treatment [26]. It has been reported that upregulation of lactate dehydrogenase-A, which plays a key role in the glycolytic pathway for regulating the conversion of pyruvate to lactate, causes resistance to paclitaxel in breast cancer treatment [27]. Furthermore, a high expression of pyruvate kinase M2 has been noted to contribute to resistance to 5-fluorouracil in patients with colorectal cancer [28]. Additionally, a high level of pyruvate dehydrogenase is linked to sorafenib-acquired resistance in hepatocellular carcinoma cells [29]. In the present study, the levels of glycolysis and mitochondrial respiration were higher in the primary CML cells than they were in the healthy cells. These data suggest that the CML cells relied heavily on glycolytic and mitochondrial functions to meet the high demands of energy production and metabolism and these effects were supported by an accelerated mitochondrial protein translation.

Tigecycline is a relatively new antibiotic that has a broad antibacterial spectrum against gram-positive and gram-negative pathogens via binding to the 30S bacterial ribosomal subunit. Interestingly, the 30S bacterial ribosome is homologous to the 28S mitochondrial ribosome in eukaryotic cells [13]. Consequently, tigecycline acts as a novel anticancer drug due to its inhibition of mitochondrial protein translation. However, whether tigecycline improves drug-resistance in CML is still unknown. Hence, we implemented a comprehensive in vitro study to evaluate the mechanism underlying the anti-leukemic effect of tigecycline. First, tigecycline inhibited the growths of CML primary cells and cell lines, including imatinib-sensitive and imatinib-resistant cells, by impairing mitochondrial biogenesis. In addition, tigecycline suppressed the activities of the mitochondrial-encoded proteins Cox-1 and Cox-2, but not the activity of the nuclear-encoded protein Cox-4. This resulted in a downregulation of mitochondrial electron transport respiratory chain and a reduction in OCR. In addition, there was mitochondrial membrane depolarization and low ROS production. As a byproduct of the electron transport chain, ROS usually increase rapidly after treatment of cells with inhibitors of the electron transport chain, such as oligomycin, rotenone, and antimycin A. We speculated that tigecycline may not inhibit electron transport chain enzymes directly in a unique mechanism distinct from other electron transport chain inhibitors do. Furthermore, tigecycline reduced the enhanced glycolytic level (ECAR) in the CML cells and showed an inhibitory effect on the CML cells, including those that were imatinib-resistant with T315I mutation. Subsequently, the tigecycline-treated CML cells were prone to apoptosis via activation of the cytochrome c/caspase-9/caspase-3 pathway. Recently, Karvela and colleagues demonstrated that knockdown of the autophagy-mediated protein ATG7 leads to decreased glycolysis [30]. Moreover, autophagy serves as a “weapon” for cancer cells to response to chemotherapy and to oppose the mechanisms of apoptosis. Therefore, we explored the role of tigecycline in autophagy in the CML cells. We observed that tigecycline induced autophagy in the CML cells by downregulating the PI3K-AKT-mTOR signaling pathway. Next, combined treatment by using tigecycline and autophagy inhibition further enhanced the sensitivity of the CML cells to tigecycline-induced cytotoxicity. Surprisingly, tigecycline showed no cytotoxicity to the normal cells because of the differences in the metabolic type and mitochondrial biogenesis between leukemic cells and their healthy counterparts. The levels of glycolysis and mitochondrial respiration in the primary CML cells were higher than the respective levels in the normal cells. Additionally, mitochondrial mass and relative mtDNA content were higher in the primary CML cells than they were in the healthy control cells.

Due to the selective anti-leukemic activity of tigecycline, the latter seems to be a promising agent for tumor treatment. However, it has been reported that, after monotherapy with tigecycline, no significant pharmacodynamic changes or clinical responses were observed in patients with refractory acute myeloid leukemia (AML) [31]. This suggests that the administration route, dose, and use of tigecycline in combination with other treatments should be explored to improve the anticancer effect of tigecycline. In this regards, most CML patients after combination treatment of tigecycline with pharmacological inhibition of autophagy may obtain efficient treatment response, even the patients with poor prognostic markers.

## Conclusions

In summary, our study showed that tigecycline inhibited the proliferation of CML primary cells and cell lines including the drug-sensitive and drug-resistant cells. Tigecycline suppressed mitochondrial functions and metabolism of CML cells and resulted in cell death by via activation of cytochrome-c/ caspase-9/ caspase-3 pathway. Furthermore, tigecycline induced autophagy by downregulation of PI3K-AKT-mTOR pathway and combination of tigecycline with inhibition of autophagy could further enhance this anti-cancer effect. Therefore, combining tigecycline use with autophagy inhibition may be a novel approach in overcoming drug resistance in CML treatment and this combination therapy should be further studied.

## Abbreviations

2-DG: 2-deoxyglucose
3-MA: 3-methyladenine
A/R: Antimucin A/ rotenone
ATG-5: Autophagy related 5
ATP: Adenosine triphosphate
CCCP: Carbonyl cyanide 3-chlorophenylhydrazone
CCK8: Cell counting kit-8
CML: Chronic myeloid leukemia
Cox-1: Cytochrome c oxidase-1
CQ: Chloroquine
ECAR: Extracellular acidification rate
FCCP: Carbonyl cyanide 4-(trifluoromethoxy)phenylhydrazone
mtDNA: Mitochdrial deoxyribonucleic acid
OCR: Oxygen consumption rate
OXPHOS: Oxidative phosphorylation
QPCR: Quantitative polymerase chain reaction
ROS: Reactive oxygen species
SDS-PAGE: Sodium dodecyl sulfate polyacrylamide gel electrophoresis
siRNA: Small interfering ribonucleic acid
TKI: Tyrosine kinase inhibitor.

## References

[1] Rowley JD (1973). Letter: A new consistent chromosomal abnormality in chronic myelogenous leukaemia identified by quinacrine fluorescence and Giemsa staining. Nature.

[2] Ben-Neriah Y, Daley GQ, Mes-Masson AM, Witte ON, Baltimore D (1986). The chronic myelogenous leukemia-specific P210 protein is the product of the bcr/abl hybrid gene. Science.

[3] Goldman JM, Melo JV (2001). Targeting the BCR-ABL tyrosine kinase in chronic myeloid leukemia. N Engl J Med.

[4] O'Hare T, Eide CA, Deininger MW (2007). Bcr-Abl kinase domain mutations, drug resistance, and the road to a cure for chronic myeloid leukemia. Blood.

[5] Desplat V, Faucher JL, Mahon FX, Dello Sbarba P, Praloran V, Ivanovic Z (2002). Hypoxia modifies proliferation and differentiation of CD34(+) CML cells. Stem Cells.

[6] Hammond EM, Asselin MC, Forster D, O'Connor JP, Senra JM, Williams KJ (2014). The meaning, measurement and modification of hypoxia in the laboratory and the clinic. Clin Oncol (R Coll Radiol).

[7] Vander Heiden MG, Cantley LC, Thompson CB (2009). Understanding the Warburg effect: the metabolic requirements of cell proliferation. Science.

[8] Viale A, Pettazzoni P, Lyssiotis CA (2014). Oncogene ablation-resistant pancreatic cancer cells depend on mitochondrial function. Nature.

[9] Vyas S, Zaganjor E, Haigis MC (2016). Mitochondria and cancer. Cell.

[10] Kroemer G, Jaattela M (2005). Lysosomes and autophagy in cell death control. Nat Rev Cancer..

[11] Klionsky DJ (2007). Autophagy: from phenomenology to molecular understanding in less than a decade. Nat Rev Mol Cell Biol..

[12] Bellodi C, Lidonnici MR, Hamilton A (2009). Targeting autophagy potentiates tyrosine kinase inhibitor-induced cell death in Philadelphia chromosome-positive cells including primary CML stem cells. J Clin Invest.

[13] Lamb R, Ozsvari B, Lisanti CL (2015). Antibiotics that target mitochondria effectively eradicate cancer stem cells, across multiple tumor types: treating cancer like an infectious disease. Oncotarget.

[14] Anderson S, Bankier AT, Barrell BG (1981). Sequence and organization of the human mitochondrial genome. Nature.

[15] Tam EW, Feigenbaum A, Addis JB (2008). A novel mitochondrial DNA mutation in COX1 leads to strokes, seizures, and lactic acidosis. Neuropediatrics.

[16] Green DR, Kroemer G (2004). The pathophysiology of mitochondrial cell death. Science.

[17] Schägger H (2002). Respiratory chain supercomplexes of mitochondria and bacteria. Biochim Biophys Acta.

[18] Liu X, Kim CN, Yang J, Jemmerson R, Wang X (1996). Induction of apoptotic program in cell-free extracts: requirement for dATP and cytochrome c. Cell.

[19] Nencioni A, Cea M, Montecucco F (2013). Autophagy in blood cancers: biological role and therapeutic implications. Haematologica.

[20] Chi KH, Wang YS, Huang YC, et al. Simultaneous activation and inhibition of autophagy sensitizes cancer cells to chemotherapy. Oncotarget 2016 July 28. [Epub ahead of print]. 10.18632/oncotarget.10873 PMC5295413 27486756

[21] Aveic S, Tonini GP (2016). Resistance to receptor tyrosine kinase inhibitors in solid tumors: can we improve the cancer fighting strategy by blocking autophagy?. Cancer Cell Int..

[22] Alers S, Löffler AS, Wesselborg S, Stork B (2012). Role of AMPK-mTOR-Ulk1/2 in the regulation of autophagy: cross talk, shortcuts, and feedbacks. Mol Cell Biol.

[23] Boya P, González-Polo RA, Casares N (2005). Inhibition of macroautophagy triggers apoptosis. Mol Cell Biol.

[24] Grimaldi A, Santini D, Zappavigna S (2015). Antagonistic effects of chloroquine on autophagy occurrence potentiate the anticancer effects of everolimus on renal cancer cells. Cancer Biol Ther.

[25] Abu-Amero KK, Kondkar AA, Azad TA, Sultan T, Kalantan H, Al-Muammar AM (2014). Keratoconus is associated with increased copy number of mitochondrial DNA. Mol Vis..

[26] Zhao Y, Butler EB, Tan M (2013). Targeting cellular metabolism to improve cancer therapeutics. Cell Death Dis..

[27] Zhou M, Zhao Y, Ding Y (2010). Warburg effect in chemosensitivity: targeting lactate dehydrogenase-A re-sensitizes taxol-resistant cancer cells to taxol. Mol Cancer..

[28] Shin YK, Yoo BC, Hong YS (2009). Upregulation of glycolytic enzymes in proteins secreted from human colon cancer cells with 5-fluorouracil resistance. Electrophoresis.

[29] Shen YC, Ou DL, Hsu C (2013). Activating oxidative phosphorylation by a pyruvate dehydrogenase kinase inhibitor overcomes sorafenib resistance of hepatocellular carcinoma. Br J Cancer.

[30] Karvela M, Baquero P, Kuntz EM (2016). ATG7 regulates energy metabolism, differentiation and survival of Philadelphia-chromosome-positive cells. Autophagy.

[31] Reed GA, Schiller GJ, Kambhampati S (2016). A Phase 1 study of intravenous infusions of tigecycline in patients with acute myeloid leukemia. Cancer Med.

